# Cluster analysis and profiling of airway fluid metabolites in pediatric acute hypoxemic respiratory failure

**DOI:** 10.1038/s41598-021-02354-4

**Published:** 2021-11-26

**Authors:** Jocelyn R. Grunwell, Milad G. Rad, Susan T. Stephenson, Ahmad F. Mohammad, Cydney Opolka, Anne M. Fitzpatrick, Rishikesan Kamaleswaran

**Affiliations:** 1 Children’s Healthcare of Atlanta, Egleston Hospital, Atlanta, GA USA; 2 Department of Pediatrics, Children’s Healthcare of Atlanta at Egleston, Division of Critical Care Medicine, Emory University School of Medicine, 1405 Clifton Road NE, Atlanta, GA 30322 USA; 3 Department of Electrical and Computer Engineering, Georgia Institute of Technology, Atlanta, GA USA; 4 Department of Biomedical Informatics, Emory University School of Medicine, Atlanta, GA USA; 5 Department of Biomedical Engineering, Georgia Institute of Technology, Atlanta, GA USA

**Keywords:** Paediatric research, Molecular medicine, Respiratory distress syndrome

## Abstract

Hierarchal clustering of amino acid metabolites may identify a metabolic signature in children with pediatric acute hypoxemic respiratory failure. Seventy-four immunocompetent children, 41 (55.4%) with pediatric acute respiratory distress syndrome (PARDS), who were between 2 days to 18 years of age and within 72 h of intubation for acute hypoxemic respiratory failure, were enrolled. We used hierarchal clustering and partial least squares-discriminant analysis to profile the tracheal aspirate airway fluid using quantitative LC–MS/MS to explore clusters of metabolites that correlated with acute hypoxemia severity and ventilator-free days. Three clusters of children that differed by severity of hypoxemia and ventilator-free days were identified. Quantitative pathway enrichment analysis showed that cysteine and methionine metabolism, selenocompound metabolism, glycine, serine and threonine metabolism, arginine biosynthesis, and valine, leucine, and isoleucine biosynthesis were the top five enriched, impactful pathways. We identified three clusters of amino acid metabolites found in the airway fluid of intubated children important to acute hypoxemia severity that correlated with ventilator-free days < 21 days. Further studies are needed to validate our findings and to test our models.

## Introduction

Pediatric acute respiratory distress syndrome (PARDS) occurs in six percent of mechanically ventilated children^1^. Although the overall mortality for children with PARDS is 17%, children with the most severe hypoxemia have a mortality of close to 33%^1^. There are no targeted therapies for children with PARDS due to a poor understanding of the underlying immunologic derangements and pathobiology. Two clinical phenotypes, a hyperinflammatory (reactive) and a hypoinflammatory (uninflamed), have been identified in adults with ARDS using latent class analysis and unsupervised hierarchal clustering^2–4^. Although ARDS phenotypes have been described using plasma cytokine targets combined with clinically available data, several adult studies have used metabolomic approaches to understand underlying ARDS heterogeneity, identify ARDS biomarkers, and discover metabolic subgroups of patients with ARDS with different mortality rates^5,6^. In children, endotype identification has focused on sepsis-triggered ARDS cytokine responses and whole blood differential gene expression using microarray technology^7–9^.

Metabolic subtyping of children with and without PARDS is a strategy to understand underlying metabolic dysregulation of PARDS and to determine whether these responses are associated with PARDS severity and fewer ventilator-free days. Stratification on metabolic and biologic responses defining a PARDS phenotype may aid in predictive and prognostic enrichment of clinical trials of targeted interventions for PARDS. The primary objective of this study is to determine whether unsupervised hierarchal cluster analysis would identify groups of children distinguished by differences in concentrations of amino acid metabolites that would be associated with the degree of hypoxemia and a primary outcome of VFD < 21 days. We hypothesized that an unsupervised approach would identify clusters of children predicted by distinguishing patterns of airway fluid amino acid metabolite concentrations that would correlate with a primary outcome of VFD < 21 days.

## Methods

### Study design and clinical characterization

Children who were endotracheally intubated within the prior 72 h for acute respiratory failure were enrolled between 2018 and 2020 at the Emory University/Children’s Healthcare of Atlanta Egleston Hospital with informed consent obtained from a parent or legal guardian prior to any procedures. The Emory institutional board approved the study protocol (IRB 00034236 and IRB 00113035). All methods were carried out in accordance with relevant guidelines and regulations (Declaration of Helsinki). Children were excluded if they were immunocompromised as previously described^10,11^. Hypoxemia thresholds were defined using the Pediatric Acute Lung Injury and Consensus Conference (PALICC) criteria and included a group of intubated children with a level of hypoxemia that did not meet the mild PARDS threshold^12^. Study participants have been described^11^. Twenty-eight-day VFD was the primary outcome and was dichotomized as VFD < 21 days and VFD ≥ 21 days^7,11,13^. Overall severity of illness was quantified using the Pediatric Risk of Mortality-III (PRISM-III) score within four hours of PICU admission^14^. Organ failures were quantified using the Pediatric Logistic Organ Dysfunction-2 (PELOD-2) score^15^.

### Sample collection and preparation

Tracheal aspirates were obtained within 72 h of intubation using inline suctioning with up to 10 ml of sterile saline lavage and sample collection in a sterile Lukens trap as previously described^10,11^. Sample were immediately placed in ice and immediately brought to lab for processing. Airway fluid was transferred to a sterile conical tube, mucus plugs and debris were dispersed by passage of the airway sample through an 18-gauge needle, followed by centrifugation in a swinging bucket rotor centrifuge at 800× G for 15 min at 4 $^\circ$C. Cell free supernatant was aliquoted into Eppendorf tubes in 250–500 µl aliquots and stored at −80 $^\circ$C until analysis.

### Amino acid metabolite analysis of airway fluid

Amino acids were measured by solid phase extraction followed by derivatization and liquid/liquid extraction (EZ:faast Kit, Phenomenex, Torrance, CA). The extraction and derivatization procedures were performed according to the manufacturer’s instructions. Samples were mixed with internal standards (homoarginine, methionine-d3 and homophenylalanine), extracted, and derivatized with propyl chloroformate. The organic phase was evaporated at room temperature under a stream of nitrogen and re-dissolved in mobile phase. Samples were analyzed using a Thermo Vanquish UHPLC coupled to a Thermo TSQ Quantis triple quadrupole mass spectrometer (Thermo Scientific, Waltham, MA). Using an autosampler at 4 °C, a volume of 1 µL was injected onto a 250 × 2.0 mm × 4 µ AAA-MS column (Phenomenex) at a flow rate of 0.25 mL/min. The column was held at 35 °C. Mobile phase A was 10 mM ammonium formate in water, and mobile phase B was 10 mM ammonium formate in methanol. Samples were separated using an 18-min gradient, from 68 to 83% of mobile phase B, with a 7-min re-equilibration between samples. The ion transfer tube and vaporizer were maintained at 275 °C and 225 °C respectively. Positive electrospray ionization mode at 5000 V was used to monitor selected reaction transitions as outlined in the EZ:faast manual. Transitions were optimized for the mass spectrometer using derivatized standards, and quantitation of amino acids was performed using TraceFinder software (Thermo Scientific).

### Cluster analysis, partial least squares-discriminant analysis, and pathway analysis

Forty-two metabolites for seventy-four patient samples were used in the cluster analysis. A total of 88 (2.9%) of the data were missing. By default, missing values were replaced by 1/5 of the minimum positive values of their corresponding variables. Metabolite concentrations were normalized by median, log transformed, and scaled by mean centering in MetaboAnalyst 5.0^16,17^. Clustering was performed using the unweighted pair group method with arithmetic mean (UPGMA)^18^. A cluster heatmap was generated using the python package seaborn^19^. Patient were visually assigned to one of three cluster groups, and a partial least squares-discriminant analysis (PLS-DA) was performed using the cluster assignment to identify metabolites contributing the most variability amongst clusters using a variable importance projection. The predictive ability of the PLS-DA model was tested using leave-one-out cross-validation^20^.

One-way analysis of variance (ANOVA) was performed to identify significant metabolites from the three clusters using the Fisher Least Significant Difference (LSD) test and a false discovery rate (FDR) of < 0.05. Significant metabolites were used in a hypergeometric over-representation analysis test to determine metabolic pathways of importance. The importance measure for topological analysis was the relative betweenness centrality measure.

### Pathway enrichment, topology analysis, and metabolic set enrichment analysis

Quantitative pathway enrichment analysis was performed using the binary classification of the primary outcome VFD < 21 days (Yes versus No) and the median normalized, log transformed, and mean center scaled metabolite concentrations using the Globaltest method. The node importance measure for topological analysis was relative betweenness centrality. The pathway impact value was calculated from pathway topology analysis. Statistical significance was determined using FDR < 0.05. Metabolic set enrichment analysis was performed using quantitative enrichment analysis using both the Kyoto Encyclopedia of Genes and Genomes (KEGG) and Small Molecule Protein Database (SMPDB) metabolic pathway associated metabolite sets in separate queries^21–23^. The enrichment analysis is performed using the R package globaltest using a generalized linear model to estimate a Q-statistic for each metabolite set, which describes the correlation between the compound concentration profiles and the primary outcome, VFD < 21 days (Yes vs. No)^24^.

### Univariate analysis of metabolites associated with VFD

Univariate analysis of metabolites for children with VFD < 21 days (Yes vs. No) was performed. Volcano plots were created and metabolites with a fold-change threshold of 2 and a FDR < 0.05 using the Wilcoxon Rank Sum Test.

### Statistical analysis

Participant characteristics were described using median and 25th–75th interquartile ranges or number and percent. A Mann–Whitney-U test was used for univariate analysis for the binary outcome VFD < 21 days (Yes vs. No). One-way analysis of variance (ANOVA) was used for continuous variables and a chi-square test was used for proportions to identify significant differences amongst the three clusters. A *p*-value < 0.05 was statistically significant.

## Results

### Clinical characteristics

There were seventy-four participants in this study. Demographic and clinical features of participants in this study have been described^11^. Clinical features stratified by degree of hypoxemia, and low (no or mild PARDS) or high (moderate or severe PARDS) severity are shown in sTable 1.

### Cluster identification

**Figure 1:**
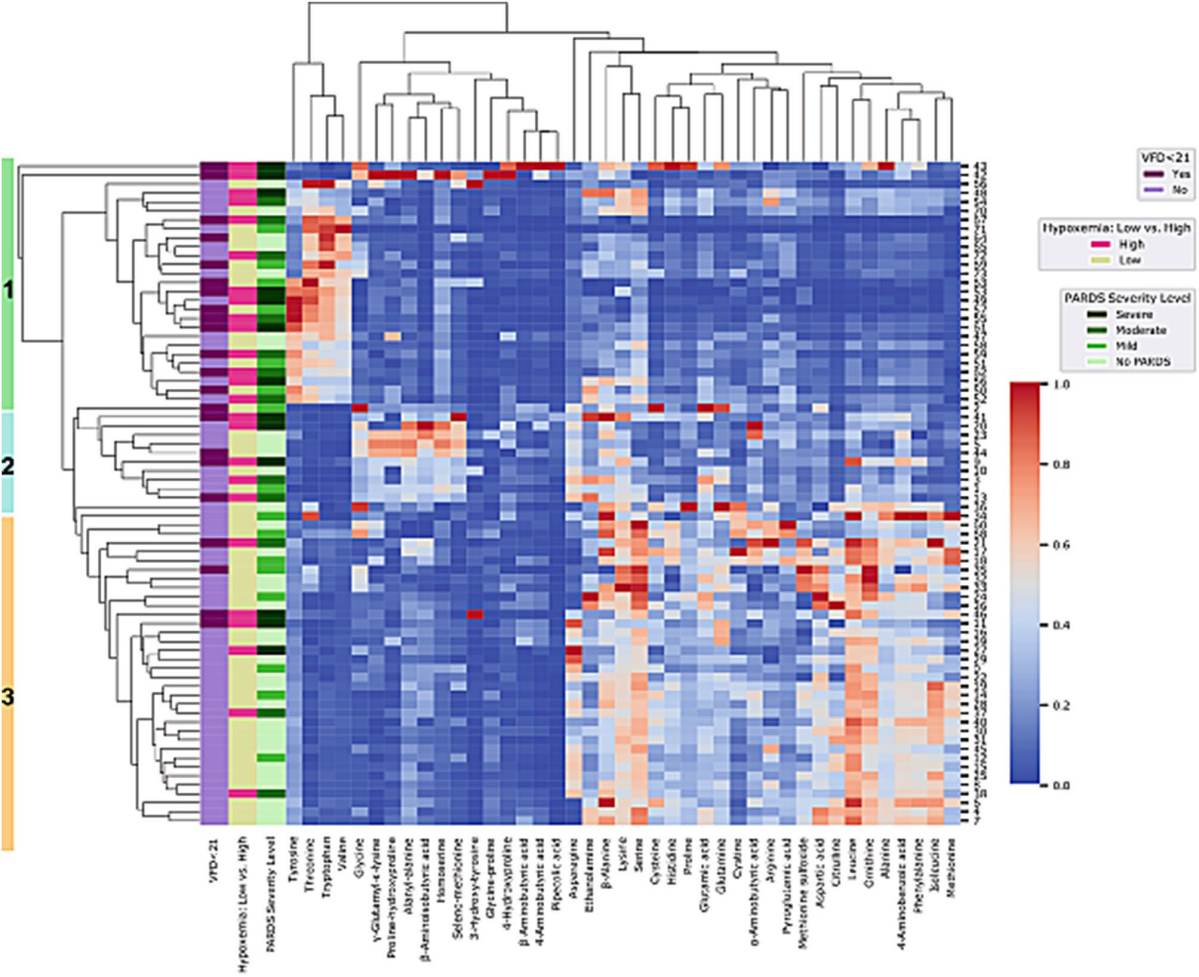
Hierarchal cluster analysis of metabolites into three patient clusters. Children with and without pediatric acute respiratory distress syndrome (PARDS) are in blue (no PARDS) and in orange (with PARDS). Children with low hypoxemia (no or mild PARDS) are in yellow and those with high hypoxemia (moderate or severe PARDS) are in magenta. Children with no, mild, moderate, or severe PARDS are shown in green from lightest (no PARDS) to darkest (severe PARDS). Children with ventilator-free days (VFD) < 21 days (more than 7 days on a ventilator or death) are in dark purple and those with VFD ≥ 21 days are in light purple. Cluster assignments are denoted by the green (cluster 1), blue (cluster 2), and orange (cluster 3) bar to the left of the participant dendrogram.

We performed an unsupervised hierarchal clustering of forty-two amino acid metabolites. Three clusters were seen by visual inspection of the heatmap (Fig. 1). Demographic and clinical characteristics of participants by cohort are show in Table 1. There were no differences in age, sex, race, ethnicity, or respiratory infection. Children in cluster 1 had more severe PARDS than children in clusters 2 and 3. Children in cluster 2 had more overall severity of illness (higher PRISM III) than children in clusters 1 and 3. Children in clusters 2 and 3 spent a longer median time of seven days versus 3.5 days on a ventilator compared with children in cluster 3 (Table 1). Increased frequency of high severity of hypoxemia (magenta bars) and a higher proportion of children with VFD < 21 days (dark purple bars) for children in clusters 1 and 2 are shown on the side of the heatmap in Fig. 1.

**Table 1:** Demographic and clinical characteristics of children by cluster.

Characteristic	Cluster			
	1	2	3	*p*-value
	*n* = 27 (36%)	*n* = 11 (15%)	*n* = 36 (49%)	
Age (years),	1.06	1.67	0.58	0.0739
Median (IQR)	(0.4, 2.5))	(0.78, 2.8)	(0.12, 1.7)	
**Sex, *n* (%)**				
Female	12 (44%)	2 (18%)	16 (44%)	0.2619
Male	15 (56%)	9 (82%)	20 (56%)	
**Race, *n* (%)**				
Black	15 (56%)	8 (73%)	15 (42%)	0.4985
White	9 (33%)	3 (27%)	16 (44%)	
Unknown	0 (0%)	0 (0%)	3 (8%)	
Multiple	3 (11%)	0 (0%)	2 (6%)	
**Ethnicity, *n* (%)**				
Hispanic or Latino	1 (4%)	0 (0%)	2 (6%)	0.7111
Non-Hispanic or Latino	26 (96%)	11 (100%)	34 (94%)	
**Severity of Illness Scores, median (range)**				
PRISM III	15 (9, 16)	23 (12, 26)	12.5 (8, 18)	0.0125
PELOD	6 (5, 7)	7 (4, 11)	6 (4, 8)	0.4333
**PARDS severity**				
Low (No/Mild)	13 (48%)	7 (64%)	30 (83%)	0.0122
High (Moderate/Severe)	14 (52%)	4 (36%)	6 (17%)	
Ventilator Days, median (Q1-Q3)	7 (3, 11)	7 (6, 19)	3.5 (2, 6)	0.0024
28 day Ventilator-free Days, median (Q1-Q3)	21 (16, 25)	21 (9, 22)	24.5 (22, 26)	0.0026
Extracorporeal Life Support, *n* (%)	3 (11%)	2 (18%)	2 (5.6%)	0.4462
**Length of Stay, median (IQR)**				
PICU (days)	8 (5, 14)	10 (9, 21)	6.5 (3.25, 10)	0.0269
Hospital (days)	11 (8, 22)	16 (10, 22)	11 (7, 16.25)	0.3668
**28-day Mortality, *n* (%)**				
Dead	2 (7.4%)	1 (9.1%)	0 (0)%	0.1256
**Respiratory Culture, *n* (%)**				
No Growth	2 (7.4%)	1 (9.1%)	9 (25%)	0.4169
Viral Only	7 (26%)	4 (36.4%)	5 (14%)	
Bacterial Growth only	3 (11%)	1 (9%)	3 (8%)	
Virus + Bacterial Co-detection	15 (55%)	5 (45%)	19 (53%)	

### Partial least squares—discriminant analysis

**Figure 2:**
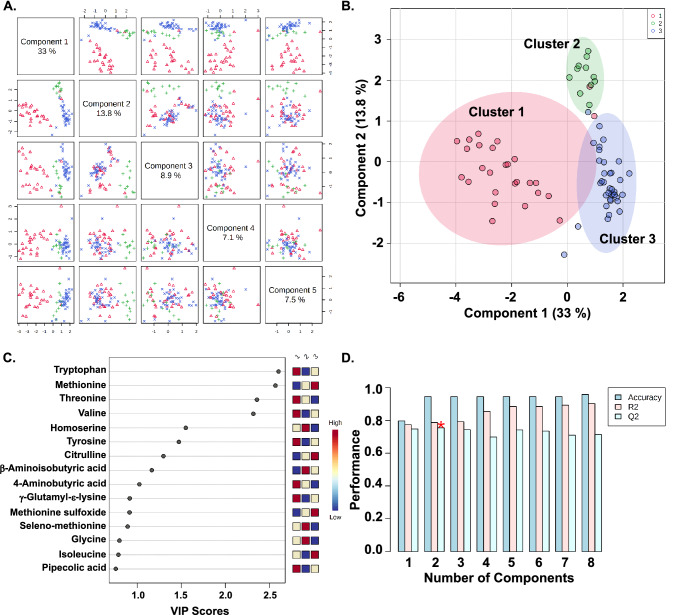
Partial least squares-discriminant analysis (PLS-DA) defined by three clusters. (**A**) Pairwise score plots for the first five components of the PLS-DA analysis. The first component explains 33% of the variability in the three groups. The second and third component explains 13.8% and 8.9% of the variability in the three groups. (**B**) The scores plot for the first two components labeled by the three clusters: cluster 1 (red), cluster 2 (green), and cluster 3 (blue). (**C**) Variable importance of projection (VIP) score plot of normalized metabolites by cluster. Higher concentrations are red. Intermediate concentrations are yellow. Lower concentrations are blue. (**D**) Values of the classification performance assessed by accuracy, goodness of fit (R2), and predictive ability (Q2) for the top six components. Two components best classify the model shown with the red asterisk using leave-one-out cross-validation.

We next used the cluster assignments to perform a partial least squares-discriminant analysis (PLS-DA) to describe the amount of variability and to identify the metabolites contributing the most to cluster variability. Variability explained by the top five components are shown in Fig. 2A. Component 1 and component 2 explain 33% and 13.8% of the variability in the clusters (Fig. 2A,B). There are two outliers in cluster 1 that are misclassified in the other clusters. Both of these patients had severe PARDS and were placed on extracorporeal life support (ECLS) for an Influenza A infection. One child was coinfected with methicillin-resistant *Staphylococcal aureus* and died after fourteen days supported by ECLS. The child who survived was supported by ECLS for nine days and was hospitalized in the pediatric intensive care unit (PICU) for 36 days.

**Figure 3:**
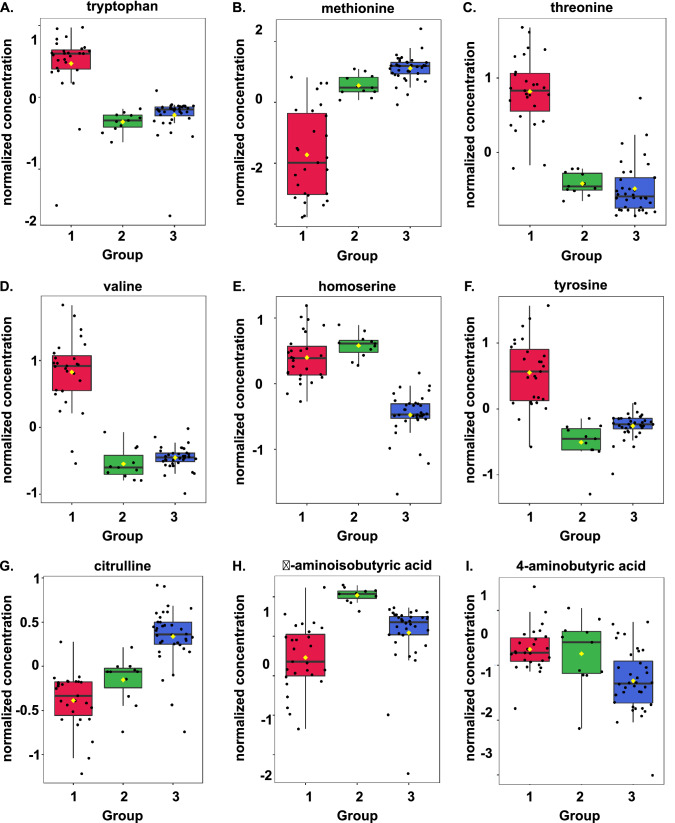
Normalized concentration of the top nine metabolites explaining the variable importance of projection (VIP) in the partial least squares-discriminant analysis (PLS-DA) by cluster or group. (**A**) Methionine, (**B**) Tryptophan, (**C**) Threonine, (**D**) Valine, (**E**) Homoserine, (**F**) Tyrosine, (**G**) Citrulline, (**H**) 4-aminobutyric acid, and (**I**) β-aminoisobutyric acid.

The metabolites contributing the most variability to each cluster are shown by the variable importance of projection (VIP) score plot of normalized metabolites by cluster (Fig. 2C). Classification performance of the PLS-DA model were assessed by accuracy, goodness of fit (R2), and predictive ability (Q2) for the top eight components (Fig. 2D). The second component best classifies the model shown with the red asterisk using leave-one-out cross-validation with an accuracy of 0.95, an R2 of 0.79, and a Q2 of 0.76 (Fig. 2D, sTable 2). Boxplots of the normalized concentrations of the top nine metabolites shown in the VIP score plot in Fig. 2C are shown by cluster (group) in Fig. 3A–I.

### Pathway analysis of clusters

Thirty out of forty-two metabolites were significantly different from each other amongst the three clusters (sTable 3). Over-representation analysis testing was used to explore the metabolic pathways important to distinguishing the three clusters. Significant metabolic pathways with an impact ≥ 0.1 and a FDR < 0.05 for children in the cohort are shown in sFig. 1. A two-cluster solution was also explored and did not reveal meaningful differences in the results (sFigs. 2 & 3).

### Pathway analysis by primary outcome

**Figure 4:**
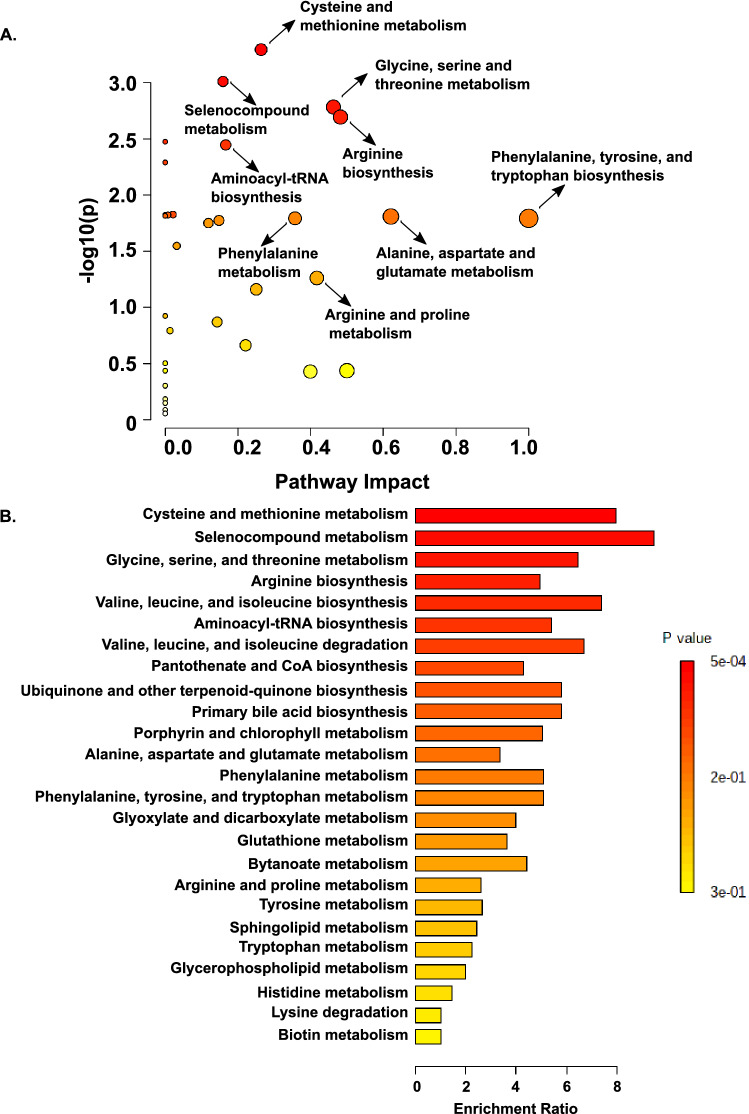
Quantitative pathway enrichment analysis using the compound concentration values to explore the metabolic differences between children with ventilator-free days (VFD) < 21 days (more than 7 days on a ventilator or death; Yes) versus those with VFD ≥ 21 days (No). (**A**) Significant metabolic pathways with an impact ≥ 0.1 for children with VFD < 21 days (Yes vs. No). (**B**) Quantitative metabolic set enrichment analysis using the Kyoto Encyclopedia of Genes and Genomes (KEGG) database for children with VFD < 21 days (Yes vs. No) using normalized metabolic concentrations from airway fluid^21–23^. Significant pathways are red and orange. The enrichment ratio is calculated as the observed hits/expected hits in the pathway.

We next performed pathway analysis combining results from a pathway enrichment analysis with a topology analysis using the KEGG database to identify the most relevant metabolic pathways involved in distinguishing children with VFD < 21 days (Yes vs. No) (Fig. 4A)^21-23^. Pathways with the highest impact and enrichment ratios include: cysteine and methionine metabolism, selenocompound metabolism, glycine, serine and threonine metabolism, arginine biosynthesis, and valine, leucine, and isoleucine biosynthesis (Fig. 4A-B). We also performed a metabolic set enrichment analysis using an alternative database, the Small Molecule Protein Database (SMPDB), using the compound concentration values to explore the metabolic differences between children with VFD < 21 days (Yes vs. No) and found similarly enriched pathways (sFig. 4A). Network analysis of the quantitative pathway enrichment analysis is shown in sFig. 4B. We performed two sensitivity analyses using only samples collected within thirty-six hours of intubation (61/74, 82% of all samples) and forty-eight hours of intubation (70/74, 95% of all samples). There were four pathways with a FDR < 0.05 for the analysis performed using only the tracheal aspirate samples collected within forty-eight hours of intubation, and these top four pathways were the same as the whole cohort (sTable 4). There were no pathways with a FDR < 0.05 for the analysis performed using only the tracheal aspirate samples collected within thirty-six hours of intubation likely due to sample size; however, the top three pathways were the same as the whole cohort (sTable5). To identify individual metabolites that were significantly different between children with VFD < 21 days (Yes vs. No) we performed a univariate analysis. Metabolites with a fold change of 2 or more and a FDR < 0.05 are shown in the volcano plot are shown (sFig. 5A). Boxplots of the normalized concentrations of the six significant metabolites alanyl-alanine, citrulline, homoserine, methionine, selenomethionine, and threonine are shown in sFig. 5B-G.

## Discussion

We used a targeted amino acid metabolite strategy along with unsupervised hierarchal clustering and PLS-DA to discover metabolic airway fluid signatures in children with acute hypoxemic respiratory failure within 72 h of endotracheal intubation. We identified three clusters in our cohort that were defined by differences in thirty metabolites with the most significant and impactful pathways including arginine biosynthesis, glycine, serine, and threonine metabolism, and cysteine and methionine metabolism using over-representation analysis. Children with no or mild acute hypoxemic respiratory failure predominated in Cluster 1; Clusters 2 and 3 were metabolically distinct endotypes made up predominantly of children with moderate or severe acute hypoxemic respiratory failure who spent a median of a week or more on invasive mechanical ventilation. A quantitative metabolic set enrichment analysis identified pathways important for distinguishing children with versus without VFD < 21 days (death or 7 or more days of invasive mechanical ventilation) identified pathway important for oxidative stress (cysteine and methionine metabolism), substrates for one-carbon metabolism (glycine, serine and threonine metabolism), branched-chain amino acids (isoleucine, leucine, and valine metabolism), and the arginine biosynthesis pathways.

There are several studies that have compared the metabolic profiles of plasma, pulmonary edema fluid, bronchoalveolar lavage fluid (BALF), and non-bronchoscopic alveolar lavage (mini BALF or mBALF) in adults with acute respiratory distress syndrome (ARDS) to healthy controls using various untargeted and targeted techniques including ^1^H-nuclear magnetic resonance (1-H NMR), gas chromatography-mass spectrometry (GC–MS) and liquid chromatography-mass spectrometry (LC–MS)^5,6,25,26^. Others have used metabolomic analyses to aid diagnosis, stratify ARDS patients by severity, and predict survival outcome^5,27–29^. Pathways distinguishing survivors from non-survivors with ARDS included glutamine and glutamate metabolism, phenylalanine, tyrosine, and tryptophan biosynthesis, and phenylalanine metabolism^6,29^. Adult endotyping studies using mBALF showed differences in lysine, arginine, tyrosine, threonine, and branched chain amino acids^6^. The findings and limitations of these adult studies have been reviewed^30^. No definitive diagnostic metabolic pattern of ARDS has emerged due to small sample sizes, variability in technique used (1H-NMR, GC–MS, LC–MS/MS) and fluid sampled (plasma, BALF, mBALF), lack of an external validation cohort, and the use of non-mechanically ventilated controls^30^.

Airway fluid obtained from readily accessible tracheal aspirate sampling of intubated children represents the metabolic derangements of the injured lung compared with plasma metabolites due to the proximity of the airway fluid to the damaged alveolar epithelial lining of the lung. We focused on amino acid based metabolic pathways because many of the predominant signatures from prior adult studies found significant differences in amino acid metabolic, degradation, and biosynthetic pathways in their analyses as previously reviewed^30^.

Increased amino acid cellular uptake and biosynthesis drive cell proliferation and control energy-generating metabolic switches by regulating glycolysis, the tricarboxylic acid (TCA) cycle, and oxidative phosphorylation (OXPHOS)^31^. Branched-chain amino acids provide acetyl-CoA and succinyl-CoA substrates for energy and reducing equivalent production via the TCA cycle^31^. Sulfur-containing amino acids, such as cysteine and methionine, and their metabolic pathways are central regulators of cellular and extracellular redox status^31^. Amino acids supply methyl and acetyl groups to accomplish post-translational modification of proteins and epigenetic modification of histones to regulate gene expression, acute immune responses and immune cell memory^31^. For example, leucine-supplied acetyl-CoA acetylates and activates mTORC1, a key intermediate in the nutrient-sensing mTOR pathway, increasing glycolysis^32^.

Amino acids support protein synthesis and metabolic programming critical for immune cell activation^31^. T cell proliferation, activation and survival depends on rapid one-carbon metabolism, or the transfer of a methyl group to various substrates, for the biosynthesis of purine nucleotides, amino acid homeostasis of glycine, serine and methionine, epigenetic maintenance, and reduction–oxidation (redox) defense. Glycine is a precursor for many essential biosynthetic pathways including glutathione, purine, creatine, and heme synthesis. When glycine supply is unavailable, serine is metabolized to glycine to fuel purine and glutathione synthesis^33^. One-carbon reactions use and create redox equivalents, such as NADPH, that are important to mitochondrial redox homeostasis^33,34^.

Cysteine is one of the amino acid precursors of glutathione, and cysteine’s metabolism and redox state is altered during acute lung injury and inflammation^35^. The uptake of cystine, the oxidized form of cysteine, is needed for T cell activation, proliferation, and DNA synthesis^31,36–38^. The requirement for sulfur amino acids increases during trauma, sepsis, and other critical illnesses^39^. T cell proliferation is impaired and activation is reduced with a deficiency of cysteine and intracellular glutathione^40^. Selenoproteins play an important role in antioxidant defense and oxidative metabolism and modulation of reactive oxygen species and inflammatory signaling pathways affecting the ability to respond to viral infections such as influenza^41,42^.

Reduced levels of arginine result in a failure of T cells to proliferate^43^. Activated neutrophils recruited to the airways of children with PARDS deplete arginine from the airway environment due to degranulate and release arginase 1^44–47^. Arginase 1 metabolism of arginine results in the synthesis of polyamines such as spermidine that limit inflammatory activity of M1-like macrophages and enhance to function of wound-healing M2-like macrophages^31^. Increased arginine metabolism by macrophages and myeloid-derived suppressor cells restricts arginine supply to T cells limiting T cell activation and interferon-γ production^31,48^. Similar to arginine, tryptophan also promotes T cell proliferation and catabolism of tryptophan by macrophages inhibits T cell activation, proliferation and cytokine production in response to infection^49^.

The pediatric acute respiratory distress incidence and epidemiology (PARDIE) study shows that many children with mild hypoxemia at onset will progress to meeting the Berlin criteria within seventy-two hours of meeting PARDS criteria^1^. It is not known how supportive treatment over the first seventy-two hours following the initiation of mechanical ventilation influences metabolic pathways in the lung environment. It is likely that some metabolites reflect treatment including the introduction of enteral and parenteral nutrition and the effect this likely has on amino acid, lipid and glucose-containing products. Significant resuscitation measures, including ventilator setting manipulation, fluid resuscitation, and initiation of antibiotics, analgosedation, and neuromuscular blockage, are likely to be ongoing within the first twenty-four to forty-eight hours following intubation and diagnosis of acute hypoxemic respiratory failure. We performed sensitivity analyses that included only samples collected within thirty-six hours and forty-eight hours after intubation. The top four pathways were the same and remained significant between the samples collect within forty-eight hours versus seventy-two hours. Due to a smaller sample size, the samples collected within thirty-six hours had the same top 3 pathways identified, but did not reach a FDR < 0.05. Severity stratification following twenty-four hours of resuscitation and stabilization may more accurately represent the true degree of lung injury due to improvement in ventilation/perfusion mismatch and lung recruitment with positive pressure ventilation^50^; however, there is little data on the temporal changes of metabolites, protein, or transcriptional biomarkers within the first days to week after onset of pediatric acute hypoxemic respiratory failure or PARDS.

There are several limitations to our single-center study. We sampled the tracheal aspirate fluid without measuring paired plasma analytes. While the majority of our samples were collected within twenty-four hours of intubation, we extended the sampling window to seventy-two hours to increase enrollment into our study. We acknowledge that the first seventy-two hours following intubation is a dynamic period where pathophysiology is evolving due to underlying the disease process, changes in ventilator settings, fluid balance, development of other organ dysfunctions, and ongoing resuscitation and clinical interventions, including blood transfusions, pressor requirements and changes in medications could confound the interpretation of our observations. Despite these limitations, in the largest multi-institutional, international epidemiologic study of PARDS, the prediction of mortality stratified by severity of hypoxemia was relatively stable from 6 to 48 h after an intubated child met PARDS criteria^1^. We did not collect serial time-points to correlate metabolite levels with clinical trajectory, and this should be a focus of future studies. While an accepted and validated non-pulmonary multiple organ dysfunction score does not exist, we acknowledge that other organ dysfunctions influence global metabolism and could impact our study findings in unknown ways despite our sampling of tracheal aspirate fluid rather than serum or plasma. We used a targeted metabolomic approach that focused on amino acids and dipeptide analytes limiting the metabolites measured. Lipid metabolites are an important component of surfactant and signaling pathways in the lung that we were unable to capture using our approach. While this is the only metabolomic study to date in intubated children with and without acute hypoxemic respiratory failure, the sample size is small, although it is larger than several adult ARDS studies^30^. There is no external validation cohort, and replication of our findings in a larger independent cohort of children at risk for and with varying severity of PARDS is needed.

In summary, we identified three patient groups using an unsupervised clustering method and explored the amino acid metabolites and pathways important to acute hypoxemic respiratory failure. We then identified metabolites and pathways that differentiated children with and without ventilator-free days < 21 days. Further studies are needed to validate our findings and to test our models.

## Supplementary Information

Supplementary Information.
