# Supplementary material for: Cluster analysis and profiling of airway fluid metabolites in pediatric acute hypoxemic respiratory failure
Source: Sci Rep. 2021 Nov 26;11:23019. doi: 10.1038/s41598-021-02354-4 (PMC8626441; doi:10.1038/s41598-021-02354-4)
Supplement: Supplementary file 1 — Supplementary Information. [file 41598_2021_2354_MOESM1_ESM.docx]

**Cluster Analysis and Profiling of Airway Fluid Metabolites in Pediatric Acute Respiratory Distress Syndrome**

Jocelyn R. Grunwell, MD, PhD, Milad G. Rad, MS, Susan T. Stephenson, PhD, Ahmad F. Mohammad, BS, Cydney Opolka, BS, Anne M. Fitzpatrick, PhD, and Rishikesan Kamaleswaran, PhD

**sFigure 1.** Overrepresentation pathway analysis of the three clusters.

**sFigure 2.** Partial least squares-discriminant analysis (PLS-DA) defined by two clusters.

**sFigure 3.** Quantitative pathway enrichment analysis of the two clusters using the Kyoto Encyclopedia of Genes and Genomes (KEGG) database.^1-3^ **A)** Summary of the pathway analysis. **B)** Pathway enrichment overview.

**sFigure 4.** Quantitative pathway enrichment analysis between children with and without ventilator-free days (VFD) < 21 days using the Small Molecule Protein Database (SMPDB). **A)** Metabolic set enrichment analysis. **B)** Network analysis.

**sFigure 5.** Univariate analysis of metabolites for children with ventilator-free days (VFD) < 21 days (Yes vs. No) using normalized metabolic concentrations from airway fluid.

**sTable 1.** Demographic and clinical characteristics of children by status and severity of pediatric acute respiratory distress syndrome (PARDS).

**sTable 2.** Partial Least Square-Discriminant Analysis leave-one-out cross validation details.

**sTable 3.** Univariate analysis of significant metabolites by cluster grouping.

**sTable 4.** Quantitative pathway enrichment analysis using the compound concentration values to explore the metabolic differences between children with ventilator-free days (VFD) < 21 days for tracheal aspirate samples collected within 48h of intubation.

**sTable 5.** Quantitative pathway enrichment analysis using the compound concentration values to explore the metabolic differences between children with ventilator-free days (VFD) < 21 days for tracheal aspirate samples collected within 36h of intubation

**sFigure 1.**

**
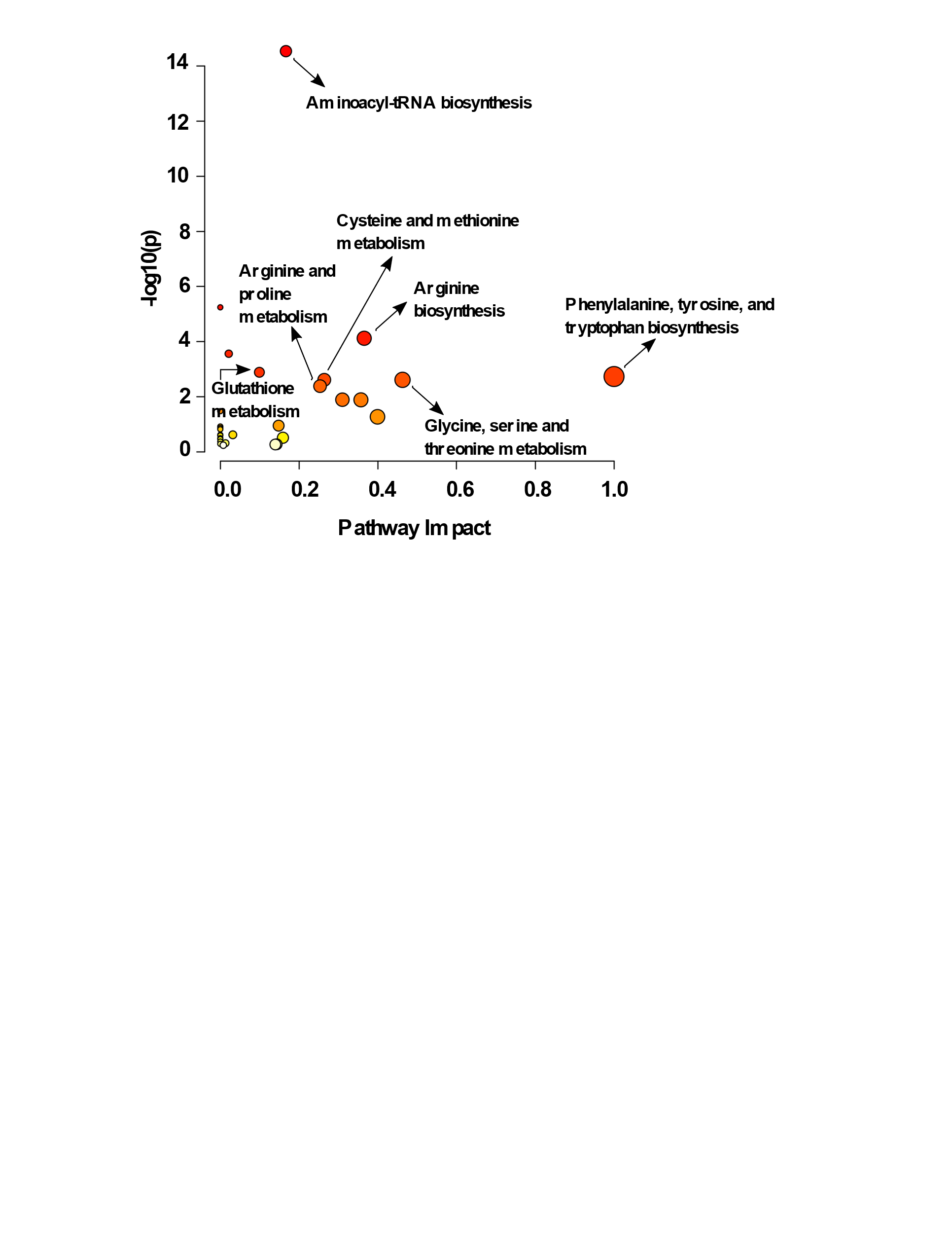
**

**sFigure 2.**

**sFigure 3.**

**sFigure 4.**

**sFigure 5.**

**sFIGURE LEGENDS**

**sFigure 1.** Pathway analysis of the thirty significant metabolites from a one-way analysis of variance (ANOVA) for the three clusters using the Fisher Least Significant Difference (LSD) test and a false-discovery rate of 0.05. Significant metabolic pathways with an impact ≥ 0.1 for children cohort are shown.

**sFigure 2.** Partial least squares-discriminant analysis (PLS-DA) defined by two clusters. Cluster 1 is the combination of clusters 1 and 2 in the three-cluster analysis **A)** Pairwise score plots for the first five components of the PLS-DA analysis. The first component explains 31.9% of the variability in the three groups. The second and third component explains 13.2% and 10.1% of the variability in the two groups. **B)** The scores plot for the first two components labeled by the two clusters: cluster 1 (red) and cluster 2 (green). **C)** Variable importance of projection (VIP) score plot of normalized metabolites by cluster. Higher concentrations are red. Lower concentrations are blue. **D)** Values of the classification performance assessed by accuracy, goodness of fit (R2), and predictive ability (Q2) for the top five components. Four components best classify the model shown with the red asterisk using leave-one-out cross-validation.

**sFigure 3.** Quantitative pathway enrichment analysis of the two clusters using the Kyoto Encyclopedia of Genes and Genomes (KEGG) database.^1-3^ **A)** Summary of the pathway analysis using normalized metabolic concentrations from airway fluid. The enrichment ratio is calculated as the observed hits / expected hits in the pathway. **B)** Pathway enrichment overview.

**sFigure 4.** Quantitative pathway enrichment analysis using the compound concentration values to explore the metabolic differences between children with ventilator-free days (VFD) < 21 days (more than 7 days on a ventilator or death; Yes) versus those with VFD ≥ 21 days (No). **A)** Quantitative metabolic set enrichment analysis using the Small Molecule Pathway Database (SMPDB) for children with VFC < 21 days (Yes vs. No) using normalized metabolic concentrations from airway fluid. Significant pathways are red and orange. The enrichment ratio is calculated as the observed hits / expected hits in the pathway. **B)** Network analysis of the quantitative pathway enrichment analysis. Each node represents a metabolite set with its color based on its p-value (red and red-orange represents a p-value < 0.05), and its size (large represents more hits) based on the number of hits to the query. Two metabolite sets are connected by an edge if the number of their shared metabolites is over 25% of the total number of their combined metabolite sets.

**sFigure 5.** Univariate analysis of metabolites for children with ventilator-free days (VFD) < 21 days (Yes vs. No) using normalized metabolic concentrations from airway fluid. **A)** Volcano plot using a fold-change threshold of 2 and a false discovery rate of 0.05 using the Wilcoxon Rank Sum Test. Four metabolites are lower in children with VFD < 21 days (Yes vs. No): alanyl-alanine, homoserine, seleno-methionine, threonine; two metabolites are higher in children with VFD < 21 days (Yes vs. No): methionine, citrulline. Isoleucine was significantly higher in children with VFD < 21 days (Yes vs. No), but did not achieve the 2-fold difference threshold. **B-G)** Normalized concentrations of metabolites with a significant 2-fold difference in level for children with VFD < 21 days (Yes vs. No). **B)** alanyl-alanine, **C)** citrulline, **D)** homoserine, **E)** methionine, **F)** seleno-methionine, and **G)** threonine.

**sTable 1. Demographic and clinical characteristics of children by status and severity of pediatric acute respiratory distress syndrome (PARDS).**

|  | PARDS Severity | |  |
| --- | --- | --- | --- |
| Characteristic | **Low**  ***n* = 50 (67.6%)** | **High**  ***n* = 24 (32.4%)** | ***p*-value** |
| Age (years),  median (IQR) | 0.84  (0.23, 2.4) | 1.1  (0.3, 2.1) | 0.6073 |
| Sex, *n* (%)  Female  Male | 21 (42%)  29 (58%) | 9 (37.5%)  15 (62.5%) | 0.7121 |
| Race, *n* (%)  Black  White  Unknown  Multiple | 26 (52%)  20 (40%)  3 (6%)  1 (2%) | 12 (50%)  8 (33%)  0 (0%)  4 (17%) | 0.1465 |
| Ethnicity, *n* (%)  Hispanic or Latino  Non-Hispanic or Latino | 2 (4%)  48 (96%) | 1 (4%)  23 (96%) | 0.9729 |
| Severity of Illness Scores,  median (range)  PRISM III  PELOD | 12 (8, 17.25)  6 (4, 7.25) | 16 (12.25, 20)  6 (5, 9.75) | 0.0534 |
| Ventilator Days, median (Q1-Q3) | 4 (2, 7) | 8 (5, 17.75) | < 0.0001 |
| Extracorporeal Life Support, *n* (%) | 0 (0%) | 7 (29%) | < 0.0001 |
| Length of Stay, median (IQR)  PICU (days)  Hospital (days) | 7 (4, 11.25)  14.5 (6.75, 17.25) | 10 (7.25, 19.5)  14.5 (11, 23.5) | 0.0026  0.0256 |
| 28-day Mortality, *n* (%)  Dead | 1 (2%) | 2 (8%) | 0.0612 |
| Respiratory Culture, *n* (%)  No Growth  Viral Only  Bacterial Growth only  Virus + Bacterial Co-detection | 9 (18%)  12 (24%)  4 (8%)  25 (50%) | 3 (12.5%)  4 (17%)  3 (12.5%)  14 (58%) | 0.7337 |

**sTable 2. Partial Least Square-Discriminant Analysis leave-one-out cross validation details.**

| Measure | Component Number | | | | |
| --- | --- | --- | --- | --- | --- |
|  | **1** | **2** | **3** | **4** | **5** |
| Accuracy | 0.7973 | 0.94595 | 0.94595 | 0.94595 | 0.94595 |
| R2 | 0.7747 | 0.78746 | 0.79197 | 0.85478 | 0.88536 |
| Q2 | 0.74816 | 0.75508 | 0.74288 | 0.69932 | 0.74229 |

**sTable 3. Univariate analysis of significant metabolites by cluster grouping**

| Metabolite Name | f.value | p.value | -log10(p) | False Discovery Rate | *post-hoc* tests^a^ |
| --- | --- | --- | --- | --- | --- |
| Valine | 115.17 | 5.17E-23 | 22.287 | 2.12E-21 | 1 - 2; 1 - 3 |
| Threonine | 100.35 | 2.04E-21 | 20.69 | 4.18E-20 | 1 - 2; 1 - 3 |
| Isoleucine | 72.167 | 7.84E-18 | 17.106 | 8.83E-17 | 3 - 1; 3 - 2 |
| Homoserine | 71.551 | 9.61E-18 | 17.017 | 8.83E-17 | 1 - 3; 2 - 3 |
| Methionine | 71.207 | 1.08E-17 | 16.968 | 8.83E-17 | 2 - 1; 3 - 1 |
| Tyrosine | 53.875 | 5.82E-15 | 14.235 | 3.98E-14 | 1 - 2; 1 - 3; 3 - 2 |
| Citrulline | 43.401 | 4.86E-13 | 12.313 | 2.85E-12 | 2 - 1; 3 - 1; 3 - 2 |
| Tryptophan | 38.123 | 5.68E-12 | 11.246 | 2.91E-11 | 1 - 2; 1 - 3 |
| Seleno-methionine | 32.597 | 9.06E-11 | 10.043 | 4.13E-10 | 2 - 1; 1 - 3; 2 - 3 |
| Ornithine | 30.65 | 2.54E-10 | 9.5957 | 1.04E-09 | 2 - 1; 3 - 1; 3 - 2 |
| Leucine | 28.022 | 1.07E-09 | 8.9706 | 3.99E-09 | 2 - 1; 3 - 1 |
| Alanyl-alanine | 20.989 | 6.89E-08 | 7.1616 | 2.36E-07 | 2 - 1; 1 - 3; 2 - 3 |
| Glycine | 20.781 | 7.86E-08 | 7.1047 | 2.48E-07 | 2 - 1; 1 - 3; 2 - 3 |
| Pyroglutamic acid | 18.202 | 4.15E-07 | 6.3816 | 1.22E-06 | 1 - 3; 2 - 3 |
| β-Aminoisobutyric acid | 17.328 | 7.44E-07 | 6.1287 | 2.03E-06 | 2 - 1; 3 - 1; 2 - 3 |
| Serine | 11.866 | 3.58E-05 | 4.4461 | 9.17E-05 | 1 - 2; 1 - 3 |
| Ethanolamine | 11.045 | 6.66E-05 | 4.1764 | 0.00016068 | 1 - 3; 2 - 3 |
| 4-Aminobenzoic acid | 10.343 | 0.00011429 | 3.942 | 0.00025362 | 2 - 1; 3 - 1; 2 - 3 |
| 4-Hydroxyproline | 10.307 | 0.00011753 | 3.9299 | 0.00025362 | 1 - 3; 2 - 3 |
| 4-Aminobutyric acid | 10.122 | 0.00013567 | 3.8675 | 0.00027812 | 1 - 3; 2 - 3 |
| Aspartic acid | 9.9857 | 0.00015086 | 3.8214 | 0.00029453 | 2 - 1; 3 - 1 |
| α-Aminobutyric acid | 9.2042 | 0.00027909 | 3.5543 | 0.00052012 | 2 - 1; 2 - 3 |
| Asparagine | 8.5326 | 0.00047767 | 3.3209 | 0.0008515 | 2 - 1; 2 - 3 |
| Phenylalanine | 7.7023 | 0.00093891 | 3.0274 | 0.0015402 | 3 - 1; 3 - 2 |
| β-Alanine | 7.702 | 0.00093913 | 3.0273 | 0.0015402 | 1 - 3; 2 - 3 |
| Methionine sulfoxide | 7.0006 | 0.0016791 | 2.7749 | 0.0026478 | 2 - 1; 3 - 1 |
| Cysteine | 6.5445 | 0.0024629 | 2.6086 | 0.0037399 | 1 - 3; |
| Arginine | 6.4811 | 0.0025983 | 2.5853 | 0.0038047 | 3 -1 ; |
| β-Aminobutyric acid | 5.914 | 0.0042111 | 2.3756 | 0.0059537 | 2 - 1; 2 - 3 |
| γ-Glutamyl-ε-lysine | 4.5863 | 0.013388 | 1.8733 | 0.018298 | 1 - 2; 1 - 3 |

**^a^** Fisher Least Significant Difference (LSD)

**sTable 4. Quantitative pathway enrichment analysis using the compound concentration values to explore the metabolic differences between children with ventilator-free days (VFD) < 21 days for tracheal aspirate samples collected within 48h of intubation**

| Pathway | False Discovery Rate | Impact |
| --- | --- | --- |
| Cysteine and methionine metabolism | 0.032477 | 0.24217 |
| Selenocompound metabolism | 0.032477 | 0.15909 |
| Glycine, serine and threonine metabolism | 0.04798 | 0.24577 |
| Arginine biosynthesis | 0.04798 | 0.48223 |
| Aminoacyl-tRNA biosynthesis | 0.064077 | 0 |
| Valine, leucine and isoleucine biosynthesis | 0.064077 | 0 |
| Primary bile acid biosynthesis | 0.064077 | 0.00758 |
| Porphyrin and chlorophyll metabolism | 0.064077 | 0 |
| Valine, leucine and isoleucine degradation | 0.064077 | 0 |
| Glyoxylate and dicarboxylate metabolism | 0.064468 | 0.10582 |
| Alanine, aspartate and glutamate metabolism | 0.064468 | 0.621 |
| Glutathione metabolism | 0.0646 | 0.11891 |
| Butanoate metabolism | 0.07807 | 0.03175 |
| Ubiquinone and other terpenoid-quinone biosynthesis | 0.10083 | 0 |
| Phenylalanine, tyrosine and tryptophan biosynthesis | 0.10083 | 1 |
| Phenylalanine metabolism | 0.10083 | 0.35714 |
| Pantothenate and CoA biosynthesis | 0.10083 | 0.02143 |
| Arginine and proline metabolism | 0.12065 | 0.41713 |
| Tyrosine metabolism | 0.40144 | 0.25057 |
| Histidine metabolism | 0.43168 | 0.22131 |
| Tryptophan metabolism | 0.45229 | 0.14305 |
| Glycerophospholipid metabolism | 0.45229 | 0.01324 |
| Nicotinate and nicotinamide metabolism | 0.53489 | 0 |
| D-Glutamine and D-glutamate metabolism | 0.53489 | 0.5 |

**sTable 5. Quantitative pathway enrichment analysis using the compound concentration values to explore the metabolic differences between children with ventilator-free days (VFD) < 21 days for tracheal aspirate samples collected within 36h of intubation**

| Pathway | False Discovery Rate | Impact |
| --- | --- | --- |
| Arginine biosynthesis | 0.06476 | 0.48223 |
| Cysteine and methionine metabolism | 0.06476 | 0.24217 |
| Glycine, serine and threonine metabolism | 0.06476 | 0.24577 |
| Primary bile acid biosynthesis | 0.06476 | 0.00758 |
| Porphyrin and chlorophyll metabolism | 0.06476 | 0 |
| Glyoxylate and dicarboxylate metabolism | 0.06476 | 0.10582 |
| Glutathione metabolism | 0.06476 | 0.11891 |
| Selenocompound metabolism | 0.06476 | 0.15909 |
| Aminoacyl-tRNA biosynthesis | 0.09237 | 0 |
| Valine, leucine and isoleucine biosynthesis | 0.10395 | 0 |
| Valine, leucine and isoleucine degradation | 0.15357 | 0 |
| Alanine, aspartate and glutamate metabolism | 0.22603 | 0.621 |
| Butanoate metabolism | 0.22603 | 0.03175 |
| Ubiquinone and other terpenoid-quinone biosynthesis | 0.30062 | 0 |
| Pantothenate and CoA biosynthesis | 0.30062 | 0.02143 |
| Arginine and proline metabolism | 0.30062 | 0.41713 |
| Nicotinate and nicotinamide metabolism | 0.30062 | 0 |
| Phenylalanine, tyrosine and tryptophan biosynthesis | 0.30062 | 1 |
| Phenylalanine metabolism | 0.30062 | 0.35714 |
| Histidine metabolism | 0.3402 | 0.22131 |
| Purine metabolism | 0.3426 | 0 |
| D-Glutamine and D-glutamate metabolism | 0.35164 | 0.5 |
| Nitrogen metabolism | 0.35164 | 0 |
| beta-Alanine metabolism | 0.43873 | 0.39925 |

**References**

1 Kanehisa, M. Toward understanding the origin and evolution of cellular organisms. *Protein Sci* **28**, 1947-1951, doi:10.1002/pro.3715 (2019).

2 Kanehisa, M., Furumichi, M., Sato, Y., Ishiguro-Watanabe, M. & Tanabe, M. KEGG: integrating viruses and cellular organisms. *Nucleic Acids Res* **49**, D545-D551, doi:10.1093/nar/gkaa970 (2021).

3 Kanehisa, M. & Goto, S. KEGG: kyoto encyclopedia of genes and genomes. *Nucleic Acids Res* **28**, 27-30, doi:10.1093/nar/28.1.27 (2000).
